# Integrin Alpha E (CD103) Limits Virus-Induced IFN-I Production in Conventional Dendritic Cells

**DOI:** 10.3389/fimmu.2020.607889

**Published:** 2021-01-27

**Authors:** Vikas Duhan, Vishal Khairnar, Simo Kitanovski, Thamer A. Hamdan, Andrés D. Klein, Judith Lang, Murtaza Ali, Tom Adomati, Hilal Bhat, Sarah-Kim Friedrich, Fanghui Li, Philippe Krebs, Anthony H. Futerman, Marylyn M. Addo, Cornelia Hardt, Daniel Hoffmann, Philipp A. Lang, Karl S. Lang

**Affiliations:** ^1^ Institute of Immunology, Medical Faculty, University of Duisburg-Essen, Essen, Germany; ^2^ Immunology in Cancer and Infection Laboratory, QIMR Berghofer Medical Research Institute, Herston, QLD, Australia; ^3^ Dana-Farber Cancer Institute, Harvard University, Boston, MA, United States; ^4^ Bioinformatics and Computational Biophysics, Faculty of Biology, University of Duisburg-Essen, Essen, Germany; ^5^ Department of Medical Laboratories, Faculty of Health Sciences, American University of Madaba, Amman, Jordan; ^6^ Department of Biomolecular Sciences, Weizmann Institute of Science, Rehovot, Israel; ^7^ Centro de Genética y Genómica, Universidad Del Desarrollo Clínica Alemana de Santiago, Santiago, Chile; ^8^ Center for Molecular Medicine Cologne, University Hospital Cologne, University of Cologne, Cologne, Germany; ^9^ Institute of Pathology, University of Bern, Bern, Switzerland; ^10^ University Medical Center Hamburg-Eppendorf, Division of Infectious Diseases, 1st Department of Medicine, Hamburg, Germany; ^11^ German Center for Infection Research, partner site Hamburg-Lübeck-Borstel-Riemse, Hamburg, Germany; ^12^ Department of Clinical Immunology of Infectious Diseases, Bernhard-Nocht-Institute for Tropical Medicine, Hamburg, Germany; ^13^ Department of Molecular Medicine II, Medical Faculty, Heinrich Heine University Düsseldorf, Düsseldorf, Germany

**Keywords:** GWAS, genome wide association screen, Itgae, CD103, vesicular stomatitis virus, IFN-I, AKT, mTOR

## Abstract

Early and strong production of IFN-I by dendritic cells is important to control vesicular stomatitis virus (VSV), however mechanisms which explain this cell-type specific innate immune activation remain to be defined. Here, using a genome wide association study (GWAS), we identified Integrin alpha-E (*Itgae*, CD103) as a new regulator of antiviral IFN-I production in a mouse model of vesicular stomatitis virus (VSV) infection. CD103 was specifically expressed by splenic conventional dendritic cells (cDCs) and limited IFN-I production in these cells during VSV infection. Mechanistically, CD103 suppressed AKT phosphorylation and mTOR activation in DCs. Deficiency in CD103 accelerated early IFN-I in cDCs and prevented death in VSV infected animals. In conclusion, CD103 participates in regulation of cDC specific IFN-I induction and thereby influences immune activation after VSV infection.

## Introduction

Type I interferon (IFN-I) is the strongest antiviral cytokine and prevents severe outcome of virus infection (1, 2). IFN-I-dependent and –independent antiviral effector programs target several stages of virus life cycle, including viral genome replication, transcription and translation (3). Most important antiviral effector molecules are the myxovirus resistance (Mx) protein, which targets incoming capsids prior to replication (4), 2,5-oligoadenylate synthetase (OAS) and RNase L degrading viral RNA (5), while Protein kinase R (PKR) and eukaryotic initiation factor 2 (eIF2α) limit translation of viral proteins (6). In addition to its antiviral activity, IFN-I is involved in several immune regulatory networks like innate immune activation, NK cell activation, CD8 T cell priming, but also immunosuppressive mechanisms i.e., interleukin-10 (IL-10) and programmed cell death ligand 1 (PD-L1) expression (7, 8). Enhanced and prolonged IFN-I activity results in over activation of the immune system which may lead to severe autoimmune disease (9).

During virus infection, signaling *via* pathogen recognition receptors (PRRs), such as Toll-like receptors (TLRs) or retinoic acid inducible gene I (RIG-I) induce IFN-I resulting in production of IFN-I (10–12). Several cell types are able to produce IFN-I, however specialized plasmacytoid dendritic cells (pDCs) produce high amounts of IFN-I, and the IFN-I induction in these cells is different from other innate cell types. In early endosomes of pDCs, contact with viral RNA or DNA leads to activation of TLR7 and/or TLR9, IRF7 activation and fast production of IFN-I (13). Conventional dendritic cells (cDCs) are also equipped with TLR7/9 and molecules of the IRF7 signaling cascade, however compared to pDCs, cDCs produce reduced amounts of IFN-I upon activation of TLR7/9 (14). Indeed, during virus infection, cDCs mainly produce IFN-I after activation of RIG-I (15). One explanation for this difference between cDCs and pDCs for IFN-I production is signaling of FcϵR1γ and DAP12, which in cDCs inhibits IRF7 activation after TLR7/9 triggering (13). More recently, the involvement of the PI3K-AKT pathway and mTOR signaling was shown to be a major innate immune regulator, which can regulate IFN-I induction and switches pDCs into high IFN-I producer (16–18). Yet, mechanisms underlying a potential differential AKT/mTOR activation linked to distinct abilities to produce IFN-I in cDCs have to be defined.

The integrin alpha E (Itgae, αE, and CD103) interact with Integrin beta 7 (ITGB7 and β7) to form heterodimer αEβ7, which is known to interact with E-cadherin (19). CD103 expression is mainly described on migrating and resident T cells and DCs in many organs such as skin, gut, lung, spleen, lymph nodes or tumors (19–21). The expression of CD103 is restricted to type I dendritic cells (CD8a+ cDC1) and absent on type II dendritic cells (CD11b+ cDC2). CD103+ cDC1 are efficient in cross presentation by sampling antigens from infected sites or tumors and migrate into the draining lymph node to mount antigen-specific T cell response. CD103+ cDC1s also cross-present self-antigens to maintain self-tolerance. CD103+ DC1s are more crucial cell type for T cell immunity than cDC2 which is necessary for host survival during respiratory viral and bacterial infections (22). Previous reports demonstrated that CD103+CD11b+ DCs respond strongly during airway infections (23–25). Further intestinal CD103+ CD11b- dendritic cells are essential to prevent severe colitis (26). This regulatory function of CD103+ CD11b- dendritic cells was linked to their ability to induce IDO1 and interleukin-18 in epithelial cells. CD103+ cDC1s support influenza replication as compared to CD103- cDC2 in the lung and are associated with dissemination of virus in the draining lymph node (27). The role of CD103 molecules on DCs is only considered to define the migratory cDC1. However, its direct role for regulating the anti-viral immunity in DC1 remains unknown.

E-cadherins are surface adhesion molecules and can modulate the PI3-kinase/Akt signaling cascade (28). In fact interaction of E-cadherin with CD103 promotes anti-tumor CTL activity (29, 30). In addition, the interaction of E-cadherin with CD103 can modulate several signaling pathways, including Wnt/β-catenin, PI3K/Akt, Rho GTPase, and NF-κB signalling (31). Such interaction will influence the function of monocytes, macrophages and DCs. Whether such modulation can influence the ability of DCs to produce IFN-I remains mainly unknown.

Here, we performed a genome-wide association screen in inbred mouse strains to determine mechanisms that regulate early virus replication and IFN-I induction in the spleen. We identified CD103 as blocker of IFN-I induction in cDCs. Mechanistically CD103 limited AKT phosphorylation and mTOR activation in cDCs.

## Methods

### Mice

Inbred mouse strains A/J (000646), AKR/J (000648), C3H/HeJ (000659), DBA/1J (000670), C57BL/6J (000664), NZW/LacJ (001058), FVB/NJ (001800), NOD/ShiLtJ (001976), CAST/EiJ (000928), BALB/cJ (000651), 129S1/SvlmJ (002448), and BTBR (002282) were purchased from Jackson. CBA/J, SJL/J and C57BL/6NJ strains were bought from Charles River. 129S6 and C57BL/6JBomTac inbred mouse strains were purchased from Taconic. *Itgae^−/−^* mice (006144) were purchased from Jackson, bred and maintained on C57BL/6J (000664) background, and littermate wild type mice used as control for experiments. *Ifnar^−/−^* mice were described previously (32) and maintained on C57BL/6 background. All animals were housed in single ventilated cages. During survival experiments, the health status of the mice was checked twice daily. Animal experiments were authorized by the Landesamt für Natur, Umwelt und Verbraucherschutz (LANUV) Nordrhein-Westfalen and in accordance with the German law for animal protection and/or according to institutional guidelines at the Ontario Cancer Institute of the University Health Network. Animals exhibiting severe symptoms of sickness or showing substantial weight loss during infection were put to death and were considered dead for statistical analysis.

### GWAS and Genphen

The GWAS was performed for 4,000,000 SNPs found in different inbred mouse strains (publicly available from http://mouse.cs.ucla.edu/mousehapmap/full.html ), and the total amount of PFU from spleen of 17 VSV infected inbred mouse lines (three individuals per line) as a quantitative trait. First, the phenotype was log10 transformed to make it conform to normality, followed by an association test between each SNP and the phenotype with EMMA as previously described (33, 34). The strength of association was summarized with p-values. A Manhattan plot for all SNPs was prepared using the SNP & Variation Suite v8.7.2 (Golden Helix, Inc., Bozeman, MT, www.goldenhelix.com).

The results obtained with EMMA were tested by a more detailed analysis with genphen (freely available at https://www.bioconductor.org/packages/genphen). To characterize the strength of association between each SNP and the phenotype, genphen computes several association metrics, including classification accuracy (how well the alleles of a given SNP are predicted from the quantitative trait), Cohen’s kappa (degree of classification accuracy above expected), Cohen’s d (effect size estimated with Bayesian inference model for testing the difference of a continuous trait between two groups (alleles of a SNP). The model was implemented in STAN (35), and is robust against outliers. It enables the computation of complete distribution of credible values for the Cohen’s d effect size. For each metric, we estimated its mean, and 99% highest density interval (HDI). The SNPs which scored high with respect to all three metrics were considered as strong associations.

### Virus, Plaque Assay, and Cell Line

VSV (Indiana strain, Mudd-Summers isolate), was originally obtained from Prof. D. Kolakofsky (University of Geneva, Switzerland). Virus was propagated on BHK-21 hamster kidney fibroblasts at a multiplicity of infection 0.01 and was plaqued onto Vero cells. VSV- EBOV was provided by Prof. Marylyn M. Addo (co-author in this paper), grown on BHK-21 cells, and quantified using Vero cells.

### Reagents and Antibodies

For flow cytometry and immunofluorescence assays, we used antibodies specific for given antigens: monoclonal antibody to VSV glycoprotein (Vi10, made in-house); CD103 (M290), CD11b (M/70) and CD16/CD32 (2.4G2) were purchased from BD Biosciences; CD103 (2E7), CD8a (53-6.7), CD11c (N418), MHC Class II (I-A/I-E) (M5/114.15.2), F4/80 (BM8), CD115 (AFS98), Ly6C (HK1.4) and Ly6G (RB6-8C5) were purchased from Thermo Fisher Scientific; IFNAR-1 (MAR1-5A3; BE0241) and isotype control IgG1 κ (MOPC-21; BE0083) were bought from Bioxcell.

Reagents used for other analysis were bought from following companies, recombinant mouse interferon alpha 4 (12115-1): PBL assay science; AKT inhibitor, GSK2141795 (HY-15965): MedChem Express; mTOR inhibitor, Rapamycin (1292): Tocris and DAPI (4’,6-diamidino-2-phenylindole, Dilactate) 2 (D3571): Thermo Fisher Scientific.

### Surface Staining and Flow Cytometry

For surface staining, samples were incubated in antibody solution prepared in FACS buffer for 30 min at 4°C. After incubation samples were washed one time with FACS buffer and processed for FACS analysis.

### Cell Preparation and Staining for Dendritic Cell Subsets Study and FACS Sorting

Spleens were injected with DMEM media containing 0.1 mg/ml DNAse (grade II, Sigma Aldrich) and 1 mg/ml collagenase D (Sigma Aldrich) and incubated at 37°C for 40 min. After incubation, spleens were minced with scissor, mixed using pipette to obtain single cell suspension, and cells were washed one time with FACS buffer. Cell suspension was passed through 100-μm strainer, and cells were treated with ACK buffer for 2 minutes to get rid of red blood cells. After one more washing step, cells were processed for antibody staining.

Cells were first blocked for Fc receptors using anti-mouse CD16/CD32 antibody (2.4G2) for 10 min at 4°C. Next, cells were stained for biotin conjugated antibodies against lineage markers CD3e (145-2C11), CD19 (6D5), N1.1 (PK136), and Ly6G (1A8) for 15 minutes. For FACS sorting, in next step, cells were incubated with anti-biotin micro beads (Miltenyi Biotec- 130-090-485) and passed through column to get rid of undesired cells. Unbounded cells were washed one time in MACS buffer and incubated with a cocktail of antibodies against CD11c, CD11b, B220 (RA3-6B2), CD8a, PDCA1 (eBio927), CD103, and fluorescent labeled Streptavidin and DAPI for 20 min. After one time washing with MACS buffer, cells were processed for FACS sorting. For normal DC subset analysis, samples were processed for antibodies staining without magnetic sorting. FACS gating strategy for different DC populations is shown in **Figure S2A**.

### Bone Marrow Derived Dendritic Cell (BMDC)

Bone marrow cells were harvested from tibiae and femurs of mice, erythrocytes were lysed with ACK lysis buffer, filtered and cultured in VLE-DMEM media (FG1445; Merck Millipore) supplemented with 5% culture supernatants containing murine GM-CSF (prepared in house from X63-GM-CSF cell line culture), or 20% of supernatant containing murine FLT3L (prepared in house from CHO FLT3-L FLAG cell culture; CHO FLT3-L FLAG cell line was kindly provided by Tim Sparwasser Institute of Infection Immunology, TWINCORE, Hannover, Germany), heat inactivated 10% fetal calf serum (10270; Gibco), 1% of penicillin, streptomycin, L-glutamine solution (G6784 Sigma), and 0.1% 50mM beta mercaptoethanol (D4551, Sigma), which constitute a complete media (CM) and cells were incubated at 37°C in a humidified atmosphere with 5% CO_2_. Bone marrow cells seeded with 3 × 10^6^ cells per petri dish (58 cm^2^) in 10 ml of CM or in 24-well plates with 2 × 10^5^ cells/well in 1 ml CM. On day 3, extra CM was added with volume equal to initial volume. On days 5–6, cells were either harvested for seeding in 24 well plates or directly treated in petri dishes.

### Western Blot

BMDCs were washed 2x with PBS and cell lysate were extracted with addition of hot SDS lysis buffer (1.1% SDS, 11% glycerol, and 0.1M Tris; pH 6.8) with 10% beta-mercaptoethanol. Total cell extracts were examined with 10% SDS polyacrylamide gel electrophoresis (SDS-PAGE) gels and transferred onto Whatman nitrocellulose membranes (GE Healthcare) according to standard techniques. Membranes were blocked in 5% bovine serum albumin (PAA Laboratories), 1% Tween-20 containing PBS for 1 hour at room temperature and were incubated with antibodies to specific antigens including phospho-Akt (Ser473) (#4060), phospho-Akt (Thr308) (#13038), Akt (Pan) (#4691), p-GSK-3β (Ser9) (#5558), GSK-3β (27C10) (#9315), p-mTOR (Ser2481) (#2974), and pan-mTOR (7C10) (#2983) which were bought from Cell Signaling Technology. The following secondary antibodies were used, anti-mouse IgG HRP linked antibody (NA931, GE Healthcare) or anti-rabbit IgG HRP linked antibody (NA934, GE Healthcare) depending on host for primary antibody. Signals were developed using ECL Western Blotting Substrate (#32209, Thermo Fisher Scientific) or SuperSignal West Femto Maximum Sensitivity Substrate (#34096, Thermo Fisher Scientific). Signals were detected with ChemiDoc imaging system (Biorad) and analysed with the Image lab software (Biorad).

### ELISA

Interferon α (IFN-α) enzyme-linked immunosorbent assay (ELISA) was performed according to the manufacturer’s protocol (PBL Interferon source).

### Quantitative RT-PCR

Total RNA was isolated using TRIzol reagent (15596, Thermo Fisher Scientific). The cDNA was synthesized with the QuantiTect reverse transcription kit (205313, Qiagen). Gene expression assay was performed using Fast SYBR Green master mix (Applied Biosystems) or TaqMan Fast Universal PCR Master mix (Applied Biosystems) with LightCycler 480 instrument (Roche). For qRT-PCR, the following primers from Qiagen were used: Interferon beta-1 (QT00249662), Interferon alpha-4 (QT01774353), Interferon alpha-2 (QT00253092), Itgae (QT00102914) and glyceraldehyde 3-phosphate dehydrogenase (GAPDH) (QT01658692). For analysis, the expression levels of all target genes were normalized against GAPDH (ΔCt). Gene expression values were calculated with the ΔΔCt method.

### Statistical Analysis

Data are expressed as means ± SEM. Student’s *t*-test was used to detect statistically significant differences between groups. Statistical analysis for survival between two groups was analyzed by log-rank (Mantel-Cox) test. The level of statistical significance was set at *P* < 0.05.

## Results

### GWAS Screen Reveals CD103 as Regulator of Viral Replication

**Figure 1:**
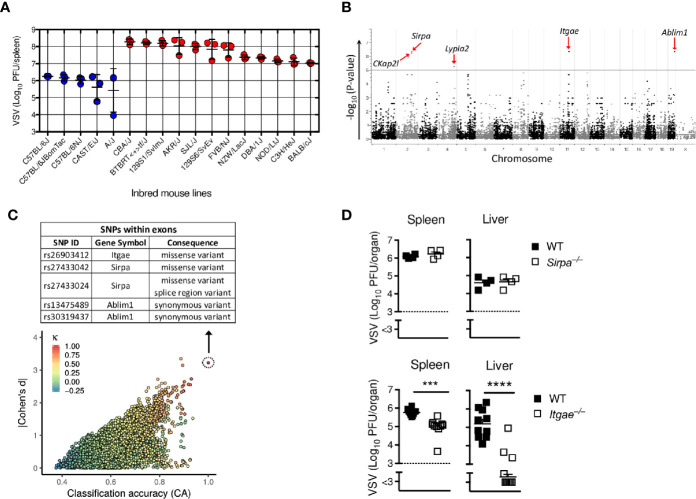
GWAS screen reveals CD103 as regulator of viral replication. **(A)** Virus titers in spleens of different inbred mouse strains which were intravenously (i.v) infected with 2x10^8^ PFU of VSV and analyzed after 8 h (*n =* 3). **(B)** Manhattan plot showing the distribution of SNPs present in each chromosome in exon regions on x-axis with its associated p-values on y-axis of the EMMA analysis based on the virus titers measured in **(A)**. Five most significantly regulated genes are highlighted with red arrows. **(C)** Plot representing distribution of reference SNPs based on classification accuracy (x-axis), Bayesian Cohen’s d (y-axis) and Cohen’s kappa (colour code) of the genphen analysis of the data in **(A)**. The list of all SNPs present in the top refSNP which is represented by dotted circle is within **Table S2**. The table depicted at top of **(C)**, shows the top SNPs located in the exon regions. **(D)** Viral titers from spleen and liver of WT or *Sirpa^−/−^* or *Itgae^−/−^* mice after i.v. infection with 2x10^8^ PFU of VSV, measured after 8h (n = 4 for WT and *Sirpa^−/−^*; n = 10 for WT and *Itgae^−/−^*, pooled from two independent experiments). Horizontal dotted lines represent the detection threshold **(D)**. Data are shown as mean ± SEM. ***P < 0.001; ****P < 0.0001 (Student’s t-test).

To determine new host factors that modulate virus replication, we performed a genome-wide association study (GWAS) in a mouse model of vesicular stomatitis virus infection (VSV). We selected 17 different commercially available inbred mouse lines, which have been genotyped for single nucleotide polymorphisms (SNPs) and are distinguished by overall 1,846,759 mutations in total of 41,017 genes (36). All 17 mouse strains were infected with 2x10^8^ PFU of VSV and after 7 hours we determined infectious replicating viral particles in the spleen. We found very diverse replication of VSV within different mouse strains (**Figure 1A**), suggesting that different variants in these strains influence replication of VSV and/or innate immune activation. EMMA-analysis of publicly available 4 million SNPs revealed that among 207,760 SNPs located in exons of coding genes, 5 top SNPs in the genes *Ckap2l, Sirpa, Lypla2, Itgae* and *Ablim1* correlated significantly with VSV replication (**Figures 1B** and **Table S1**). To test this first statistical survey using an independent approach, we applied the genphen method (described in material and methods), which classifies all SNPs to their corresponding phenotype. Using this approach, we found SNPs in 28 known different genes which could explain the differences in VSV replication (**Figure 1C** and **Table S2**). Among these 28 genes, 2 genes, Itgae and Sirpa had a missense variant (**Figure 1C**). Because these two genes were also among the top hits of the EMMA analysis, we considered them as most probable for explaining the differences found in the different inbred mouse strains. To validate the direct role of Sirpa and Itgae on VSV replication, we infected *Sirpa^−/−^* and *Itgae^−/−^* mice with VSV and analyzed viral replication after 8 hours. Viral titers were not affected in *Sirpa^−/−^* mice, but were reduced in spleen and liver of *Itgae^−/−^* mice (**Figure 1D**). From these results we concluded that the missense SNP rs26903412 in the Integrin alpha-E (*Itgae*, CD103) gene on chromosome 11 position 73,115,640 (**Supplementary Figure 1**) could explain the different VSV replication in the different mouse strains and, more important, that CD103 is one important regulator, which influence early VSV propagation.

### CD103 Limits Early Interferon Production and Enhances Susceptibility to Virus Infection

**Figure 2:**
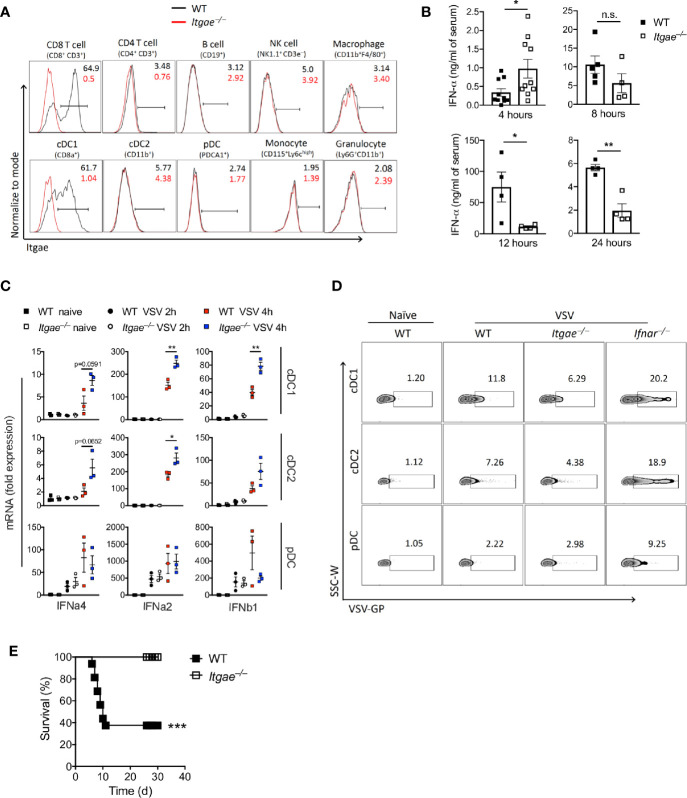
CD103 limits early Interferon production and enhances susceptibility to virus infection. **(A)** Representative FACS plots for CD103 expression on different splenic immune cells of naïve WT and *Itgae^−/−^* mice (n = 4). Values in the plots represents the frequency of cells within CD103 positive bar. **(B)** Total IFN-α in serum of WT and *Itgae^−/−^* mice, which were infected with 2x10^8^ PFU of VSV and at various time points as indicated (n = 3-10). **(C)** Gene expression data determined by RT-PCR in various FACS sorted DC subtypes from spleens of WT and *Itgae^−/−^* mice which were left untreated or were infected i.v with 2x10^9^ PFU of VSV for 2h or 4h (n = 3). **(D)** Representative FACS plots showing staining for VSV glycoprotein (GP) on splenic DC subtypes of naïve WT, and WT, *Itgae^−/−^* and *IFNAR^−/−^* mice which were infected i.v with 2x10^9^ PFU of VSV for 4h (n = 3). **(E)** Survival of WT and *Itgae^−/−^* mice, which were infected with 2x10^7^ PFU of VSV (n = 14-16). Data are shown as mean ± SEM or Kaplan Meier graph. n.s. not significant; *P < 0.05; **P < 0.01; ***P < 0.001; (Student’s t-test for B and C, and Mentel-Cox survival test for C).

Next, we aimed to analyze the mechanisms leading to different viral replication in the absence of CD103. We considered that early innate immune activation and induction of antiviral programs was one likely explanation for differences in viral replication. Indeed, CD103 was mainly expressed in conventional DCs (**Figure 2A**), suggesting this cell type was mainly responsible for different VSV replication in *Itgae^−/−^* mice and inbred mice with the missense SNPs. Since fast innate immune activation and production of IFN-I is most effective to block VSV replication, we considered that IFN-I response may be modulated by CD103. During VSV infection first pDCs produce IFN-I which is hardly systemically detectable, but still important for viral control (37). In a second line of defense, conventional dendritic cells produce systemically measurable IFN-I (37). To get insights on the role of CD103 on IFN-I induction we first analyzed systemic IFN-alpha in WT and *Itgae^−/−^* mice. *Itgae^−/−^* mice showed increased systemic IFN-alpha already 4 hours after infection (**Figure 2B**). At later time points IFN-alpha levels were similar or even enhanced in WT mice (**Figure 2B**). This suggests that either other innate immune cells such as pDCs produce higher amounts 4 hours after VSV infection, or that in addition to pDCs, conventional DCs produce IFN-I already 4 hours after VSV infection. To get insights we infected WT and *Itgae^−/−^* mice with VSV and then sorted splenic cDC1s, cDC2s and pDCs and analyzed their capacity to produce IFN-I. After 4 hours of VSV infection, pDCs of WT versus *Itgae^−/−^* mice showed similar levels of IFN-I mRNA expression (**Figure 2C**). Therefore, we conclude, that IFN-I mRNA expression by pDCs is not significantly influenced by CD103 expression (**Figure 2C**). In contrast cDC1s and cDC2s of WT versus *Itgae^−/−^* mice showed enhanced levels of IFN-I mRNA expression (**Figure 2C**), which suggests that absence of CD103 enhances the capacity of these cell type to produce IFN-I, albeit the overall increase of IFN-I mRNA expression in pDCs compared to cDC1 and cDC2 was much higher in infected versus uninfected mice. CD103 expression on cDC2 was absent in naïve state (**Figure 2A**), however it was induced upon VSV infection (**Supplementary Figure 2B**) which explains the enhanced IFN-I response in cDC2s after VSV infection (**Figure 2C**). The enhanced IFN-I induction in cDCs could not be explained by enhanced replication of VSV in these cells, as VSV replication was reduced in *Itgae^−/−^* cells (**Figure 2D** and **Supplementary Figure 2C**). Next, we analyzed whether the different IFN-I induction and viral replication impacted on virus control. Survival experiments revealed enhanced survival of *Itgae^−/−^* mice (**Figure 2E**), suggesting an important role of CD103 during VSV infection. Together these data show that lack of CD103 enhanced IFN-I production in conventional dendritic cells, which impacts on outcome of the infection.

### CD103 Limits IFN-I Response in Bone Marrow Derived Dendritic Cells

**Figure 3:**
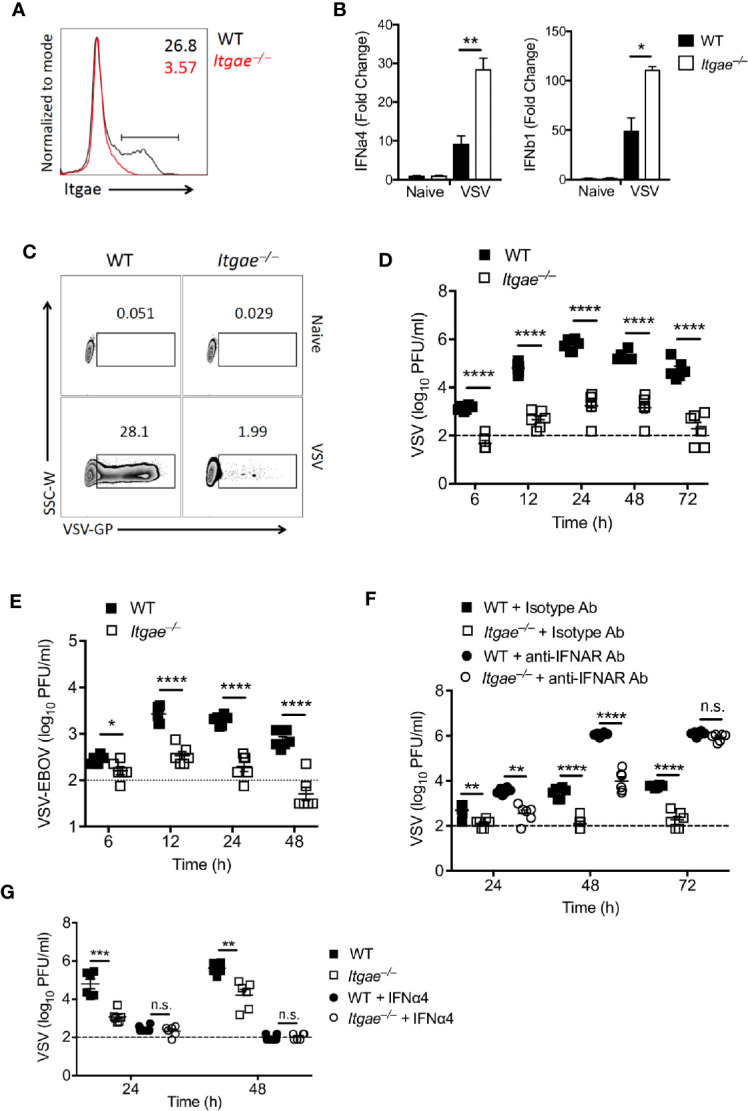
CD103 limits IFN-I response in bone marrow derived dendritic cells. **(A)** Representative FACS plot showing CD103 expression on MHCII^+^CD11c^+^ cells from naïve BMDC culture from WT or *Itgae^−/−^* mice (n = 3). Values in the plots represents the frequency of cells within CD103 positive bar. **(B)** Expression of *Ifna4* and *Ifnb1* determined by RT-PCR in WT and *Itgae^−/−^* BMDC which were left untreated or were treated with VSV (MOI = 1 for 3 h, n = 3). **(C)** Representative FACS plot showing VSV-GP staining from naïve or VSV treated (MOI-0.01 for 24h) BMDC generated from WT or *Itgae^−/−^* mice (n = 3). **(D, E)** BMDC generated from WT and *Itgae^−/−^* mice (n = 6) incubated with VSV-WT (MOI = 1, D) or VSV- EBOV (MOI = 1, E) and after 1h media was replaced with virus free fresh complete media and virus titers measured in supernatant at indicated time points. **(F)** Virus titers in supernatants of WT and *Itgae^−/−^* BMDC, which were treated with anti-IFNAR antibody (40µg/ml) or isotype control antibody (40µg/ml), measured after infection with VSV (MOI = 0.001, n = 6). **(G)** Virus titers in supernatants of WT and *Itgae^−/−^* BMDC, which were treated with IFNa4 (100U/ml), measured after infection with VSV (MOI = 0.01, n = 6). Data are shown as mean ± SEM. n.s. not significant; *P < 0.05; **P < 0.01; ***P < 0.001; ****P < 0.0001 (Student’s t-test).

To validate our hypothesis that CD103 regulates IFN-I response in cDCs, we generated dendritic cells by culturing bone marrow cells (BMDCs) in presence of GMCSF which are most close to cDCs (38). BMDCs expressed CD103 (**Figure 3A** and **Supplementary Figure 3A**). To test their ability to produce IFN-I in response to VSV, we infected BMDC cultures with VSV and measured IFN-I in RT-PCR. In line with the data from ex vivo analyzed cDCs, we found elevated IFN-I induction in *Itgae^−/−^* BMDCs at early time point (**Figure 3B**). Again, enhanced IFN-I induction could not be explained by enhanced viral replication, as VSV replication was strongly reduced in *Itgae^−/−^* BMDCs (**Figure 3C** and **Supplementary Figure 3B**). Also, production of infectious virus was strongly reduced in in *Itgae^−/−^* BMDC cultures (**Figure 3D**). Culture of bone marrow (BM) cells in presence of GMCSF result in a mixture of myeloid derived cells including cDCs, monocytes and macrophages (39). To confirm our phenotype specificity to cDCs, we cultured BM cells in presence of GMCSF and FLT3L which mainly derive cDCs differentiation (**Supplementary Figure 3C**). We could find similar suppression pattern for virus replication in *Itgae^−/−^* BMDCs from theses cultures (**Supplementary Figure 3D**). Further, we excluded that enhanced virus replication in presence of CD103 was due to a direct interaction of VSV-GP with CD103, as the recently licensed Ebola vaccine VSV-EBOV showed similarly reduced replication in *Itgae^−/−^* BMDCs cultures (**Figure 3E**). Rather, we considered that the reduced VSV replication in BMDC cultures was due to enhanced IFN-I induction in cultures of *Itgae^−/−^* BMDCs. Indeed, blockage of interferon-α/β receptor (IFNAR) enhanced replication of VSV in *Itgae^−/−^* BMDCs to WT levels after 72h of infection, although in earlier time points CD103 still accelerated VSV propagation (**Figure 3F**). Vice versa treatment with IFN-a4 limited VSV replication in WT BMDCs to levels of *Itgae^−/−^* BMDCs (**Figure 3G**). In conclusion we found that intrinsic expression of CD103 on dendritic cells limited induction of IFN-I and thereby was responsible at least partially for accelerated VSV replication.

### CD103 Modulates AKT Activation and mTOR Signaling

**Figure 4:**
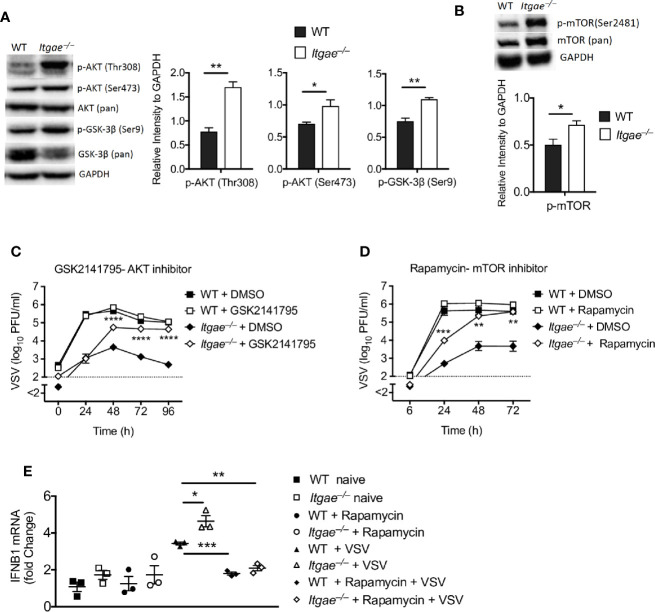
CD103 modulates AKT activation and mTOR signaling. **(A)** Representative western blots for naïve WT and *Itgae^−/−^* BMDC detected with antibodies to phospho-AKT (Thr308), phospho-AKT (Ser473), AKT (pan), phospho-GSK-3β (Ser9), GSK-3β (pan) and GAPDH. Graphs show the quantifications for p-AKT (Thr308), p-AKT (Ser473), and phospho-GSK-3β (Ser9) from naïve WT and *Itgae^−/−^* BMDCs (n = 3). **(B)** Representative western blots for naïve WT and *Itgae^−/−^* BMDC detected with antibodies to p-mTOR (Ser2481) and mTOR (pan). Graph shows the quantifications for p-mTOR (Ser2481) from naïve WT and *Itgae^−/−^* BMDCs (n = 3). **(C)** Virus titers at different time intervals after virus infection in supernatants of WT and *Itgae^−/−^* BMDC, which were treated with or without the AKT inhibitor GSK2141795 (4500 nM) for 24h and infection with VSV (MOI = 0.01, n = 6). Data shown is pooled from two independent experiments. **(D)** Virus titers at different time intervals after virus infection in supernatants of WT and *Itgae^−/−^* BMDCs, which were treated with or without the mTOR inhibitor Rapamycin (200 nM) for 24h and infection with VSV (MOI = 0.01, n = 3). **(E)** Expression of *Ifnb1* determined by RT-PCR in WT and *Itgae^−/−^* BMDC which were treated with or without rapamycin (200nM) for 24h and infected with VSV or left without infection for 3h (MOI = 1). Data are shown as mean ± SEM. n.s. not significant; *P < 0.05; **P < 0.01; ***P < 0.001; ****P < 0.0001 (Student’s t-test).

In a next step, we addressed the molecular mechanisms leading to high IFN-I production in *Itgae^−/−^* BMDCs. IFN-I is produced upon activation of pattern recognition receptors which recognize viral nucleic acids and positively regulated by various molecules such as AKT/mTOR (18, 40), glycogen synthase kinase 3 (GSK3), NF-κB, histone deacetylase 3 (HDAC3) and tyrosine-protein phosphatase non-receptor type 22 (PTPN22) (41). To get insights we generated BMDCs from WT and *Itgae^−/−^* mice and analyzed for known pathways that regulate IFN-I signaling. Interestingly we found increased phosphorylation of AKT in *Itgae^−/−^* BMDCs (**Figure 4A**). Next, we analyzed for GSK-3β which is a prime target of AKT in downstream signaling and get degraded upon phosphorylation. We detected higher phosphorylation of GSK-3β and reduced total GSK-3β protein level (**Figure 4A**). One downstream target of AKT is mTOR (42). mTOR was shown to be one major contributor to IFN-I production in pDC (18). Indeed, phosphorylated mTOR was overexpressed in *Itgae^−/−^* BMDCs when compared to WT (**Figure 4B**). In line with these findings, inhibition of AKT and mTOR signaling enhanced VSV replication in *Itgae^−/−^* BMDCs, but not in WT BMDCs (**Figures 4C, D**), suggesting that enhanced AKT/mTOR activation is one crucial parameter underlying the reduced virus replication observed upon CD103 deficiency. Further to confirm the direct role of mTOR inhibition with rapamycin in regulation of IFN-I response, we treated BMDCs with rapamycin and analyzed early IFN-I response upon VSV infection (**Figure 4E**). As expected, rapamycin treatment inhibited the virus induced early IFN-I response in *Itgae^−/−^* BMDCs similar to the level of WT BMDCs. These findings suggest that CD103 inhibits IFN-I response in AKT/mTOR dependent manner which promotes virus replication in DCs.

## Discussion

Here, we found, using a mouse model of vesicular stomatitis virus (VSV) infection, that Integrin alpha-E (*Itgae*, CD103) modulated IFN-I induction in cDCs. Mechanistically CD103 limited AKT signaling and mTOR activation, one pathway which is essential for early IFN-I response. *In vivo*, lack of CD103 resulted in enhanced early IFN-I production and improved virus control and survival during VSV infection.

pDCs are specialized IFN-I producing cells. Especially during VSV infection, production of IFN-I by pDCs a few hours after infection is essential for virus control (37, 43). Conventional dendritic cells (cDCs) are also equipped with TLR7. However, in contrast to pDCs, cDCs produce hardly any IFN-I after uptake of VSV into TLR7 containing endosomes, and only extensive replication of VSV in cDCs leads to RIG-I activation and IFN-I induction (37, 44, 45). Our data indicate that expression of CD103 is one factor, which explains this difference between pDCs and cDCs. Lack of CD103 in cDCs accelerated the production of IFN-I. The enhanced IFN-I production in CD103-deficient cDCs could not be explained by extensive replication of VSV in cDCs, as we found limited replication of VSV in CD103-deficient cDCs. From our data it remains unexplained whether the enhanced IFN-I production during VSV infection was specific for either TLR7 or RIG-I. As we found increased mTOR signaling in CD103-deficient cDCs, we consider that both pathways might be affected by CD103. cDC1 are known to express higher level of TLR3 than TLR7. For this study we used VSV, which is a single stranded RNA virus. VSV can activate several innate receptors including TLR4, TLR7, and RIG-I. Therefore, we cannot conclude on the specific role of CD103 in TLR3 signaling. Further studies have to be done to clarify this point.

We consider it likely that the enhanced IFN-I induction is not specific for VSV. This would suggest that cDCs are hyper-reactive in terms of IFN-I production, which might accelerate autoimmune disease. Indeed, it was reported previously that mice lacking CD103 start to develop spontaneous inflammatory skin disease after 6 months of their age on 129/Sv x BALB/c background (46). Whether this autoimmunity is linked to over-activated cDCs and is dependent on IFN-I remains unknown so far. Further studies have to be undertaken to address the possible role of CD103, IFN-I signaling and autoimmunity.

We found that high CD103 expression in the spleen is mainly restricted to naïve CD8^+^ T cells and CD8a^+^ DC (cDC1). CD103 expression on cDC1 modulated IFN-I and promoted virus replication. We have not identified how CD103 influences IFN-I induction and whether certain ligands modulate IFN-I response in cDCs. E-cadherin is one interaction partner of CD103 which can modulate functional activity in CD103^+^ CD8 T cells (30). Therefore, we could consider that the interaction of E-cadherin with CD103 influences IFN-I induction in DCs.

Van Prooyen et al. published that during fungal infection CD103+ conventional DCs are the major producer of IFN-I and IFN-gamma in the lungs of wild-type mice (25). While these data may look contradictory to our work, the authors did not directly analyze the role of CD103 in IFN-I expression, but rather used expression of CD103 to identify the specific dendritic subset. We could speculate that either, these cell types have limited interaction with E-cadherin or that absence of the CD103 signaling in their system might still enhance the production of IFN-I.

We found that CD103 affected also replication of VSV-EBOV which might have an impact on the outcome of vaccination with VSV-EBOV, which was recently licensed as the first Ebola vaccine and is currently administered large scale in the ongoing Ebola outbreak in the Democratic Republic of Congo. In humans SNPs in the CD103 genes are known, however whether these modulate the interaction of CD103 with E-cadherin and thereby modulate IFN-I responses remains to be studied. If indeed SNPs in CD103 would modulate the binding to E-cadherin such SNPs would accelerate IFN-I response and adaptive immune activation after vaccination. Because VSV- EBOV can persist in joints after vaccination (47), and therefore can lead to side effects, such SNPs could correlate with reduced incidence of side effects. More data have to be generated to shed light in this interaction.

In conclusion we found that CD103 blocks IFN-I induction in cDCs *via* modulation of AKT and mTOR signaling.

## Data Availability Statement

The raw data supporting the conclusions of this article will be made available by the authors, without undue reservation.

## Ethics Statement

The animal study was reviewed and approved by Landesamt für Natur, Umwelt und Verbraucherschutz (LANUV) Nordrhein-Westfalen.

## Author Contributions

VD and VK designed and performed the experiments, analyzed data, and wrote the manuscript. SK executed GWAS and genphen analysis, and wrote and corrected the manuscript. TH performed some *in vitro* and *in vivo* experiments and analyzed the data. AK was involved in initiation, analysis, and discussion of GWAS. JL helped for performing FACS sorting and RT-PCR for DC subsets. MA, TA, and HB performed immunohistology for VSV- EBOV, serum IFN ELISA and helped in other assays. S-KF and FL helped for *in vitro* BMDCs culture experiments. PK was involved in the design of the genetic approach, data discussion and identifying CD103, and corrected the manuscript. AF was involved in data discussion. MMA provided VSV- EBOV vaccine, reviewed the manuscript, and discussed the data. CH was involved in experiment design and data discussion. DH initiated and discussed the GWAS and Genphen study, and wrote and corrected the manuscript. PL discussed the data, study design and the GWAS approach and corrected the manuscript. KL initiated, proposed, and organized the study, wrote, and finalized the manuscript. All authors contributed to the article and approved the submitted version.

## Funding

This study was funded by Deutsche Forschungsgemeinschaft (DFG) grants LA1419/7-1, LA1419/10-1, LA1558/5-1, and 715 SI1558/3-1. This study was further supported by the Sonderforschungsbereich SFB974, the Transregio TRR60, and the Research Training Groups RTG1949/1 and RTG2098, the Jürgen Manchot Foundation (MOI) and FONDECYT 1180337.

## Conflict of Interest

The authors declare that the research was conducted in the absence of any commercial or financial relationships that could be construed as a potential conflict of interest.

## Supplementary Material

Supplementary Figure 1 Association between mouse genotype and splenic virus replication of inbred mouse strains. Table showing the details for a top SNP in the Itgae gene associated with virus replication correspondence to its genotype and average virus particles (log_10_ transformed, n = 3) per spleen in different strains. ‘A’ represents Adenine and ‘G’ represents Guanine.

Supplementary Figure 2 CD103 inhibits IFN-I response in cDCs. **(A)** Representative gating strategy for DC subsets in the spleen of WT mouse. **(B)** Gene expression data determined by RT-PCR in various FACS sorted DC subtypes from spleens of WT and *Itgae^−/−^* mice which were left untreated or were infected i.v with 2x10^9^ PFU of VSV for 2h or 4h (n = 3). **(C)** Graphs showing the staining for VSV glycoprotein (GP) on splenic DC subtypes from spleens of naïve WT, and WT, *Itgae^−/−^* and *IFNAR^−/−^* mice which were infected i.v with 2x10^9^ PFU of VSV for 4h (n = 3-5). Data are shown as mean ± SEM. *P < 0.05; **P < 0.01 (Student’s t-test).

Supplementary Figure 3 CD103 inhibits IFN-I response in BMDCs. BMDCs derived from GMCSF **(A, B)** or GMCSF plus FLT3L **(C, D)** cultures were kept without infection for control or infected with VSV. **(A)** FACS plot showing the frequency of CD103 cells in MHC-II+ cells from naïve BMDC (n = 3). **(B)** Frequency of VSV-GP staining from VSV treated (MOI-0.01 for 24h) BMDC generated from WT or *Itgae^−/−^* mice (n = 3). **(C)** FACS plot showing the frequency of CD103+ cells in total MHC-II+ cells from naïve BMDC (n = 3). **(D)** Representative FACS plot and graph showing VSV-GP staining from naïve or VSV treated (MOI-0.01 for 24h) BMDC generated from WT or *Itgae^−/−^* mice (n = 3). Data are shown as mean ± SEM. *P < 0.05; **P < 0.01; ***P < 0.001; and ****P < 0.0001 (Student’s t test).

Supplementary Table 1 List of SNPs associated with virus replication analyzed by EMMA. This table includes all SNPs present in known genes along with different variants with a cut off p-value (-log_10_) = 3 which are associated with virus replication in different inbred mouse lines from **Figure 1B**.

Supplementary Table 2 List of SNPs present in top reference SNP analyzed by genphen. This table includes all SNPs present in top refSNP which is denoted by dotted circle in **Figure 1C**.
