# Supplementary figures and images for: Integrin Alpha E (CD103) Limits Virus-Induced IFN-I Production in Conventional Dendritic Cells
Source: Front Immunol. 2021 Jan 27;11:607889. doi: 10.3389/fimmu.2020.607889 (PMC7873973; doi:10.3389/fimmu.2020.607889)

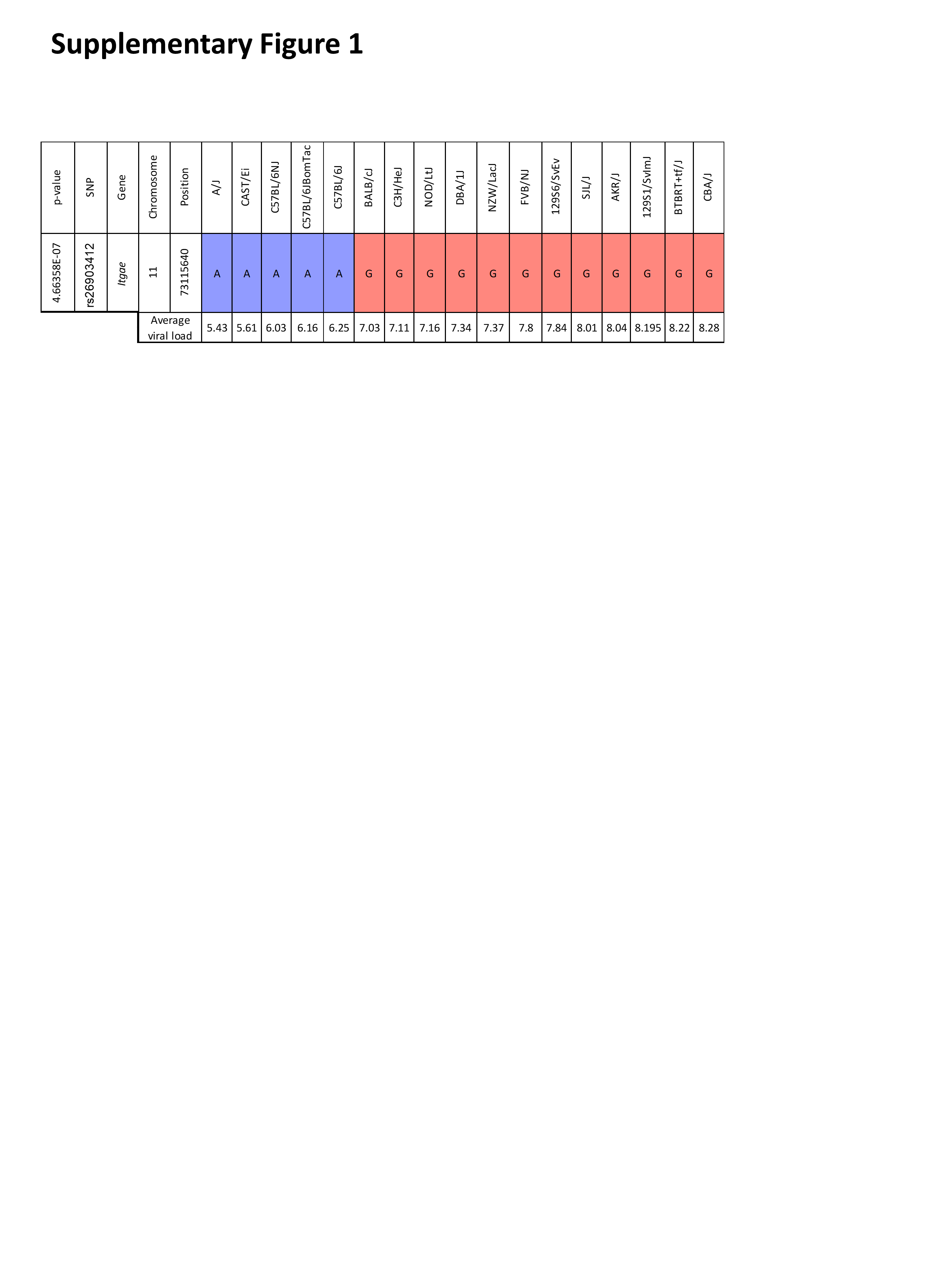

Supplement: Supplementary Figure 1 — Association between mouse genotype and splenic virus replication of inbred mouse strains. Table showing the details for a top SNP in the Itgae gene associated with virus replication correspondence to its genotype and average virus particles (log10 transformed, n = 3) per spleen in different strains. ‘A’ represents Adenine and ‘G’ represents Guanine. [file Image_1.tif]

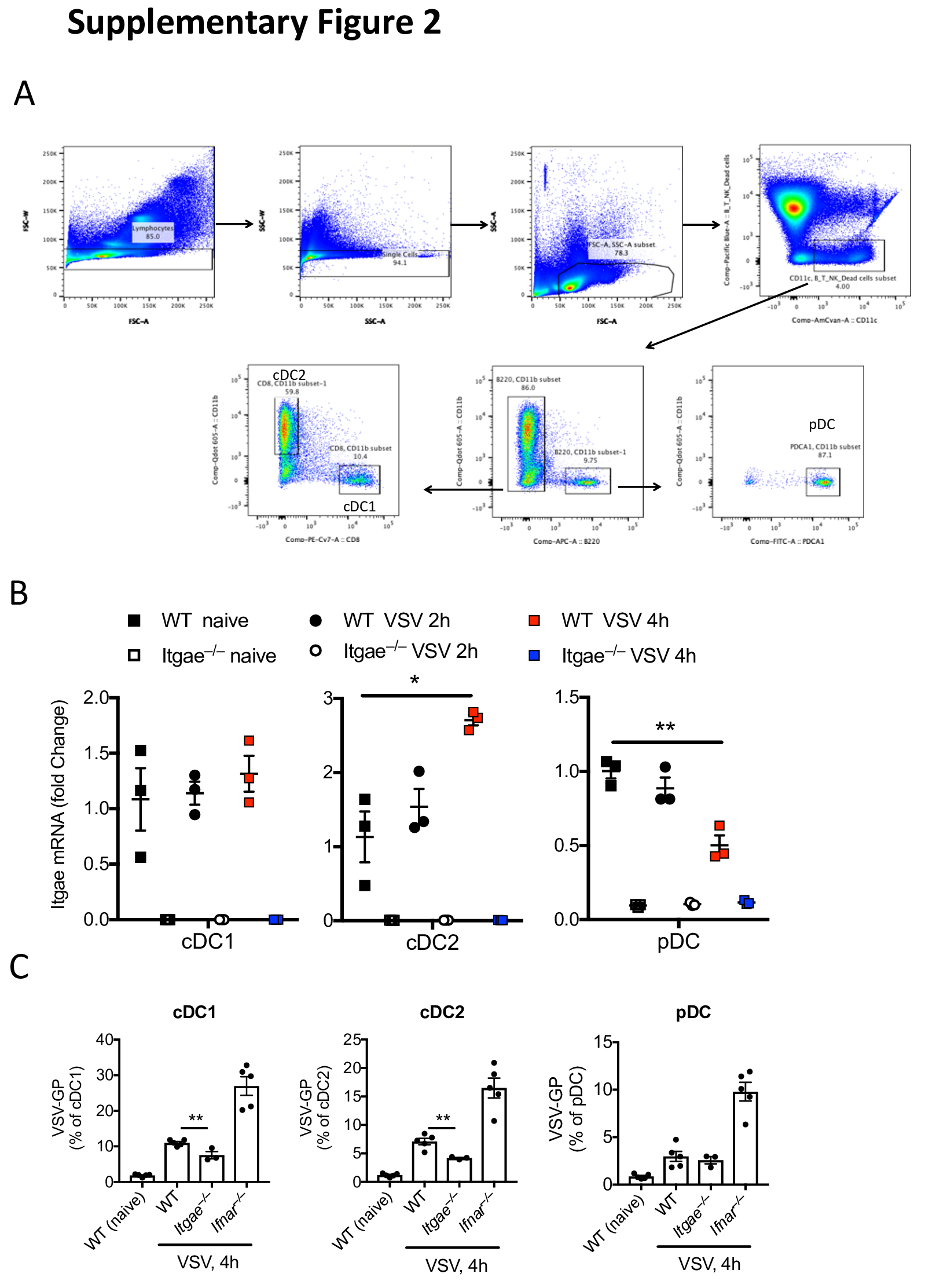

Supplement: Supplementary Figure 2 — CD103 inhibits IFN-I response in cDCs. (A) Representative gating strategy for DC subsets in the spleen of WT mouse. (B) Gene expression data determined by RT-PCR in various FACS sorted DC subtypes from spleens of WT and Itgae−/− mice which were left untreated or were infected i.v with 2x10^9^ PFU of VSV for 2h or 4h (n = 3). (C) Graphs showing the staining for VSV glycoprotein (GP) on splenic DC subtypes from spleens of naïve WT, and WT, Itgae−/− and IFNAR−/− mice which were infected i.v with 2x10^9^ PFU of VSV for 4h (n = 3-5). Data are shown as mean ± SEM. *P < 0.05; **P < 0.01 (Student’s t-test). [file Image_2.tif]

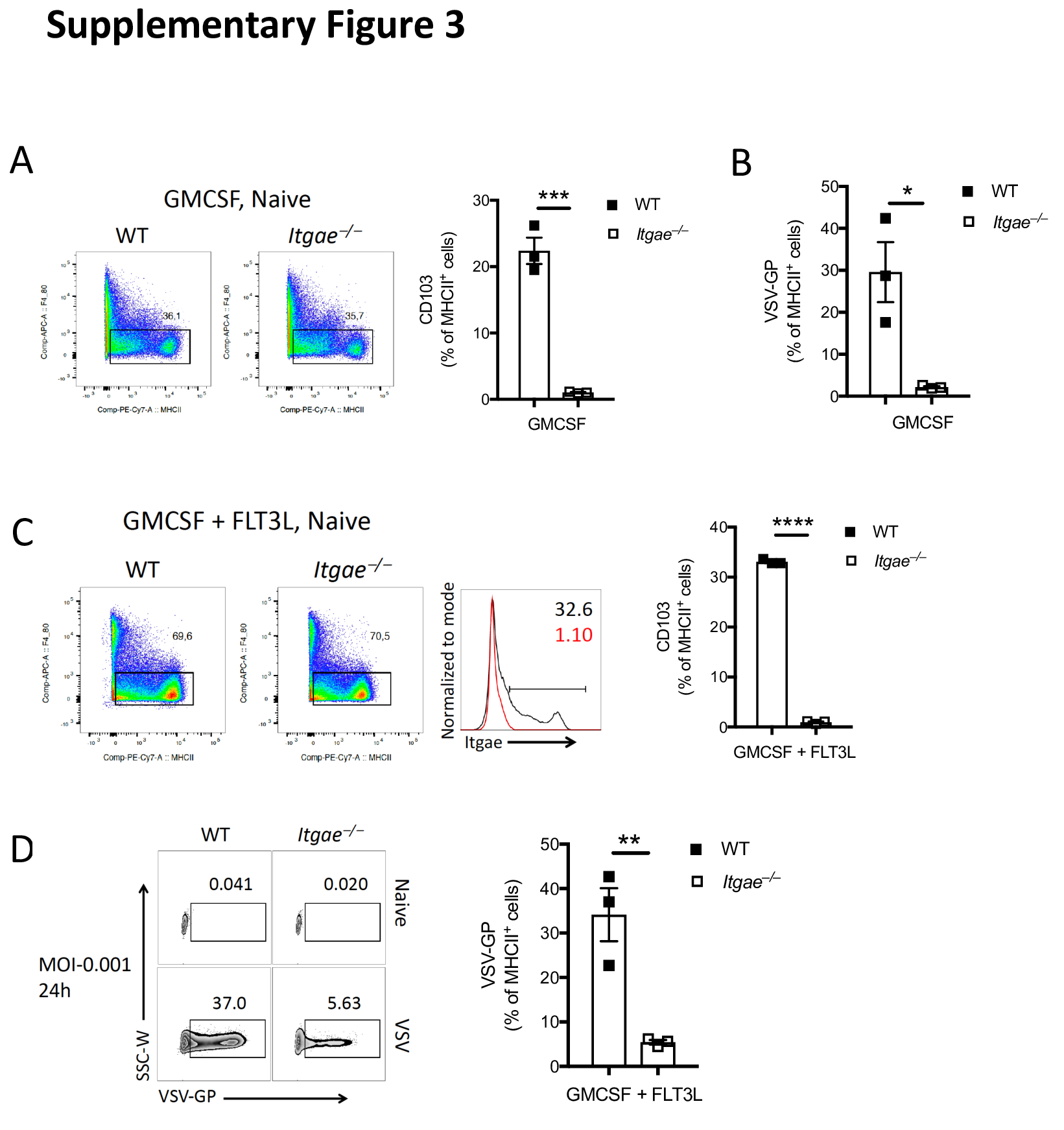

Supplement: Supplementary Figure 3 — CD103 inhibits IFN-I response in BMDCs. BMDCs derived from GMCSF (A, B) or GMCSF plus FLT3L (C, D) cultures were kept without infection for control or infected with VSV. (A) FACS plot showing the frequency of CD103 cells in MHC-II+ cells from naïve BMDC (n = 3). (B) Frequency of VSV-GP staining from VSV treated (MOI-0.01 for 24h) BMDC generated from WT or Itgae−/− mice (n = 3). (C) FACS plot showing the frequency of CD103+ cells in total MHC-II+ cells from naïve BMDC (n = 3). (D) Representative FACS plot and graph showing VSV-GP staining from naïve or VSV treated (MOI-0.01 for 24h) BMDC generated from WT or Itgae−/− mice (n = 3). Data are shown as mean ± SEM. *P < 0.05; **P < 0.01; ***P < 0.001; and ****P < 0.0001 (Student’s t test). [file Image_3.tif]
